# Supplementary material for: Intrinsic functional neuron-type selectivity of transcranial focused ultrasound neuromodulation
Source: Nat Commun. 2021 May 4;12:2519. doi: 10.1038/s41467-021-22743-7 (PMC8097024; doi:10.1038/s41467-021-22743-7)
Supplement: Supplementary file 1 — Supplementary Information [file 41467_2021_22743_MOESM1_ESM.pdf]

Supplementary Materials

of

Intrinsic Functional Neuron-type Selectivity of Transcranial Focused Ultrasound Neuromodulation

Kai Yu[1,2]†, Xiaodan Niu[1]†, Esther Krook-Magnuson[3], Bin He[1*]

1. Department of Biomedical Engineering, Carnegie Mellon University
2. Department of Biomedical Engineering, University of Minnesota
3. Department of Neuroscience, University of Minnesota

† These authors contributed equally to this work.

* Correspondence:

Bin He, PhD

Department of Biomedical Engineering

Carnegie Mellon University

5000 Forbes Avenue, Pittsburgh, PA 15213, USA

e-mail: bhe1@andrew.cmu.edu

Supplementary Figure 1

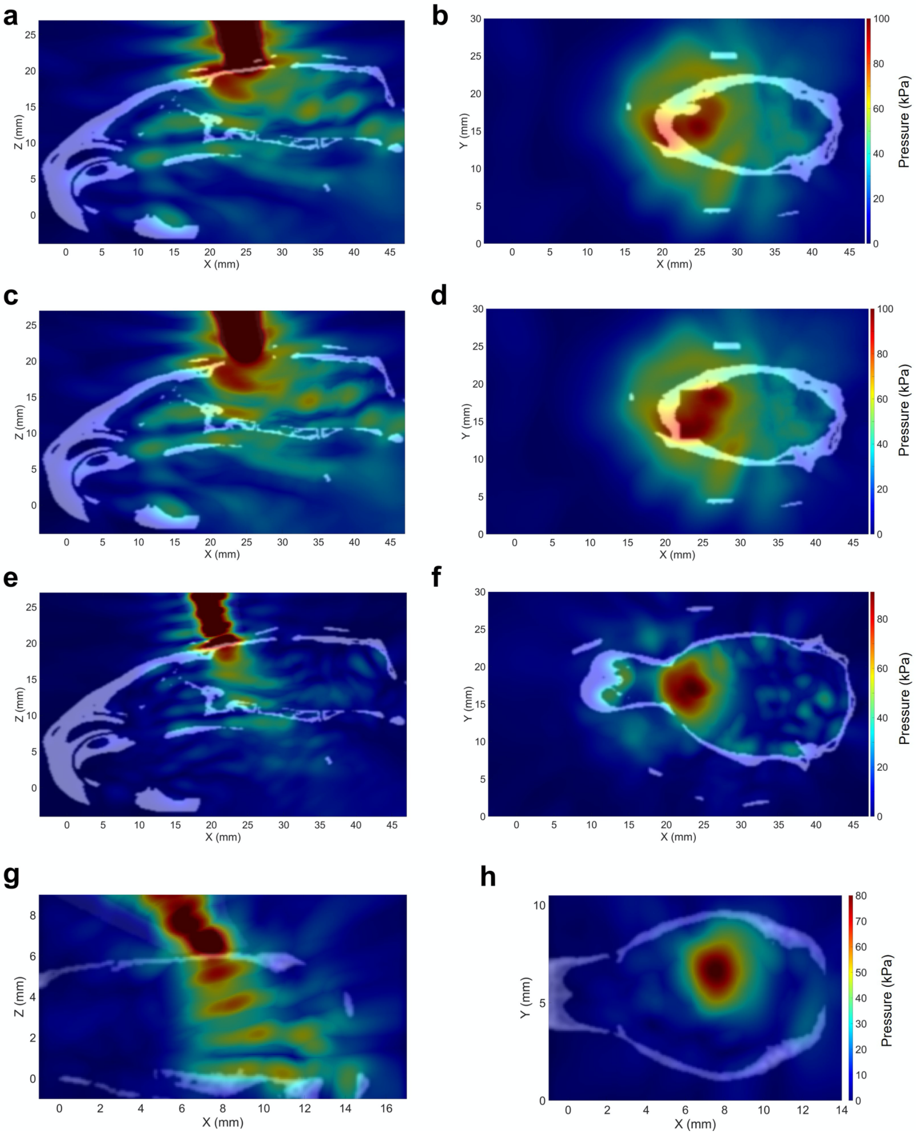

Supplementary Figure 1. 3D Computer Simulations for Mapping the Ultrasound Pressure Field inside the Skull Cavity of Rodent Models, Related to Figs. 1, 2 and 7, Discussion and Methods.

(**a-b**) Pressure field mapping for 220 kHz ultrasound fundamental frequency (f_0), 100 sinusoidal cycle per pulse (about 454 µsec tone burst duration), 100 kPa source pressure.
(**c-d**) Pressure field mapping for 220 kHz f_0, 10 msec tone burst duration, 100 kPa source pressure. This combination of ultrasound parameters were used in [1]. The top local cranial bone along the ultrasound incidence pathway is removed as described in [1]. By removing the cranial bone, the standing wave is observed as increased.
(**e-f**) Pressure field mapping for 350 kHz f_0, 2 msec tone burst duration, 420 kPa source pressure. This combination of ultrasound parameters was applied to rat somatomotor area in [2]. This increased f_0 is able to reduce the extensiveness of ultrasound pressure field within the rat skull cavity.

(**g**-**h**) Pressure field mapping within a full mice skull for 500 kHz f_0, 200 µsec tone burst duration, 4500 Hz PRF with 30-degree ultrasound incidence angle guided with the collimator for mice (see details in Methods). The extensive ultrasound field with standing wave pattern can be observed in (g). Source data are provided in the Source Data file.

Note:

Numerical Estimations of Transcranial Ultrasound Fields inside Full Rodent Skull Models

Ex-vivo pressure mapping inside a full enclosed rodent skull is challenging to achieve with a mechanically-scanning needle hydrophone. Thus, in order to examine the pressure distributions in the rodent skull cavity, numerical simulations were conducted to estimate the ultrasound wave distribution in the skull. More field estimations using other reported stimulation parameters are selected from the literature[1,2]. As seen in Supplementary Fig. 1a-f, at lower ultrasound fundamental frequencies (f_0), the maximum amplitude of the standing waves is significantly amplified compared to the 500 kHz frequency used in our study (Fig. 2d-e). This large band, high amplitude standing waves is one of the factors, if not the only one, to cause the confounding auditory side-effects due to the use of lower fundamental frequencies. These numerical simulations were conducted with the k-Wave MATLAB toolbox[3] based on the micro-CT images of a Wistar rat head (see Methods for details). Supplementary Fig. 1g-h depict the potential standing wave pattern forming inside a full mice skull cavity using an f_0 of 500 kHz pulsed at 4500 Hz ultrasound pulse repetition frequency (PRF). The incidence angle is 30 degree (the same as illustrated in Fig. 7a) in this simulation study on this mice model.

Supplementary Figure 2

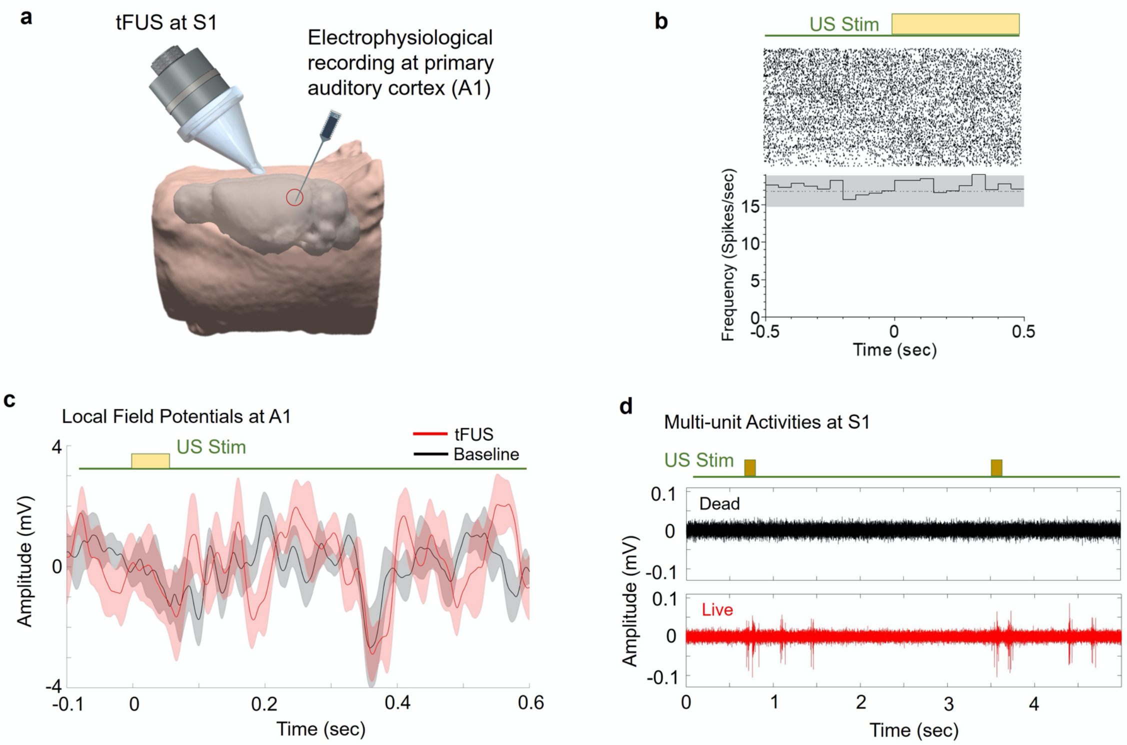

Supplementary Figure 2. Spatial Specificity of tFUS Induced Brain Activations, Related to Figs. 1 and 3.

(**a**) tFUS is administered at S1, while the electrophysiological recordings take place at the auditory cortex (A1).
(**b-c**) No statistically significant brain activations in terms of neuronal spiking activity (b) and local field potentials (LFP) (C) can be observed at A1 evidenced through the temporal pattern of action potentials and the comparison between the tFUS and the baseline conditions. Data in (c) are shown as the mean±s.e.m..
(**d**) Multi-unit activity waveforms of recorded stimulation response in live (red trace) and dead (black trace) animal under tFUS stimulation at PRF=3000 Hz. Source data are provided in the Source Data file.

Note:

Control Studies of A1 Recordings When tFUS Targets at S1

Supplementary Fig. 2a-c show the results from primary auditory cortex (A1) recording (setup is illustrated in Supplementary Fig. 2a) with tFUS stimulation directed to primary somatosensory cortex (S1). The result in Supplementary Fig. 2b presents the raster plot across all tFUS trials and peri-stimulus time histogram PSTH plot averaged through all trials for the spiking rates. In this

setup, a train of tFUS pulses are delivered during sonication durations of 500 msec (we intentionally extended the sonication duration by 7.5 times for this control study). During each trial, 150 pulses of tFUS at PRF of 300 Hz with DC at 50% and 100 cycles per pulse at fundamental frequency of 500 kHz is delivered. The panel below the raster plot shows the PSTH over 493 trials. Shaded region illustrates the 95% confidence interval around the mean firing rate shown in the dotted line. Supplementary Fig. 2c shows the local field potential at A1, while using the same sonication duration as the original 67 msec. Through a non-parametric statistical test, no significant difference was observed between the tFUS and Baseline conditions.

Another comparison is illustrated in Supplementary Fig. 2d in regard of the multi-unit activity (MUA) recordings from an *in-vivo* living brain (in red) with sonication time-locked neuronal spiking activity and from the dead brain (in black) once the animal gets euthanized. Two ultrasound stimulation events are presented in Supplementary Fig. 2d.

Supplementary Figure 3

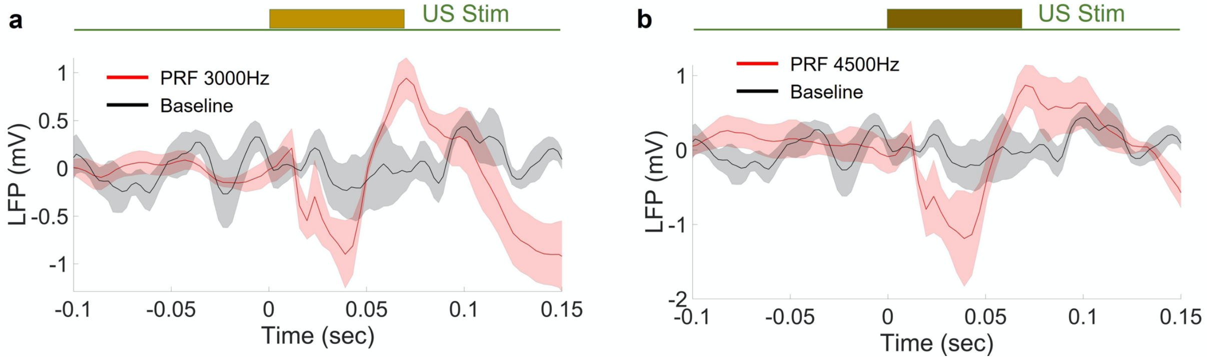

Supplementary Figure 3. LFPs in Response to tFUS Stimulation, Related to Fig. 3, Results and Discussion.

In wild-type rats (N = 5) without chemical deafening, minimally delays of the LFP activation respect to the tFUS using PRF of 3000 Hz (a) and 4500 Hz (b) are observed. The LFP are presented with the mean value (solid line) and standard error of the mean (shaded areas). Note: the reduced time window from -0.1 sec to 0.15 sec in both panels is to better illustrate the minimal delay of LFP activation respect to the onset of tFUS administered to the wild-type rats. Source data are provided in the Source Data file.

Supplementary Figure 4

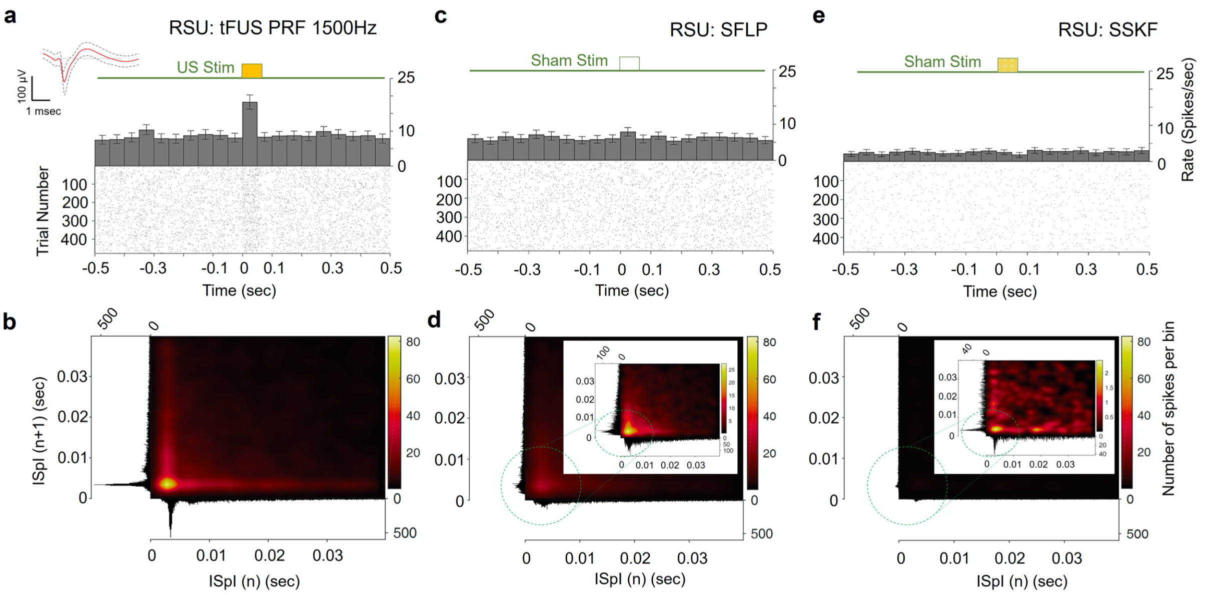

Supplementary Figure 4. Temporal Dynamics of a Regular Spiking Unit (RSU) in Responding to Administered Ultrasound and Sham Conditions, Related to Fig. 3.

All figures presented are from the same neuron under different conditions. The peri-stimulus time histograms (PSTHs, bin size: 50 msec, n = 478 trials for each time bin) and raster plots of the spiking unit (IP duration mean: 850 µsec; AHP duration mean: 1850 µsec, waveform depicted as an inset in (**a**)) responding to a tFUS condition (illustrated Fig. 1a) with PRF of 1500 Hz (**a**), and sham conditions used as a negative control (**c**) in which the ultrasound aperture was active but flipped away from the animal subject (SFLP, illustrated as in Fig. 1e) and a positive control (**e**) in which the ultrasound was directed to an anterior part of the skull (SSKF, illustrated as in Fig. 1f). (**a**, **c**, and **e**) PSTH data are representative firing rates of the same neuron from one rat subject. Data shown as the mean±95% confidence interval.

The time histograms and return plots in (**b, d** and **f**) compared the differences of the first and second order statistics of inter-spike waiting time using a bin size of 0.1 msec. The different colormaps are intended to visually separating FSU in Supplementary Fig. 5 from RSU in this figure, and the specific colormap used here is for a better visualization of the temporal features. Source data are provided in the Source Data file.

Supplementary Figure 5

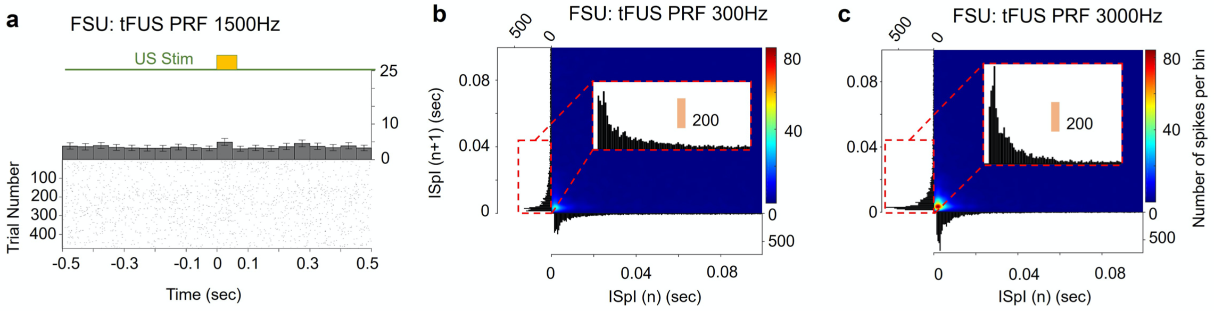

Supplementary Figure 5. The Spiking Statistics of a Fast Spiking Unit (FSU), Related to Fig. 3.

(**a**) The peri-stimulus time histogram (PSTH, n = 477 trials for each time bin) of one fast-spiking unit (IP duration mean: 700 μsec; AHP duration mean: 600 μsec) recorded from an anesthetized rat in response to the tFUS conditions with PRF of 1500 Hz. The temporal dynamics are computed across 477 trials, with each trial lasting 2.5 sec. The applied ultrasound conditions are described in Table 1 (see Methods). Data are shown as the mean±95% confidence interval in the PSTH.
(**b**-**c**) The return plots of the FSU in response to tFUS conditions with the PRF of 300 and 3000 Hz, corresponding to Fig. 3f and g, respectively. The insets show the enlarged time histograms of the inter-spike interval. Notice the change of spiking pattern in response to these two conditions. The temporal dynamics are computed across 478 trials, with each trial lasting 2.5 sec. Source data are provided in the Source Data file.

Supplementary Figure 6

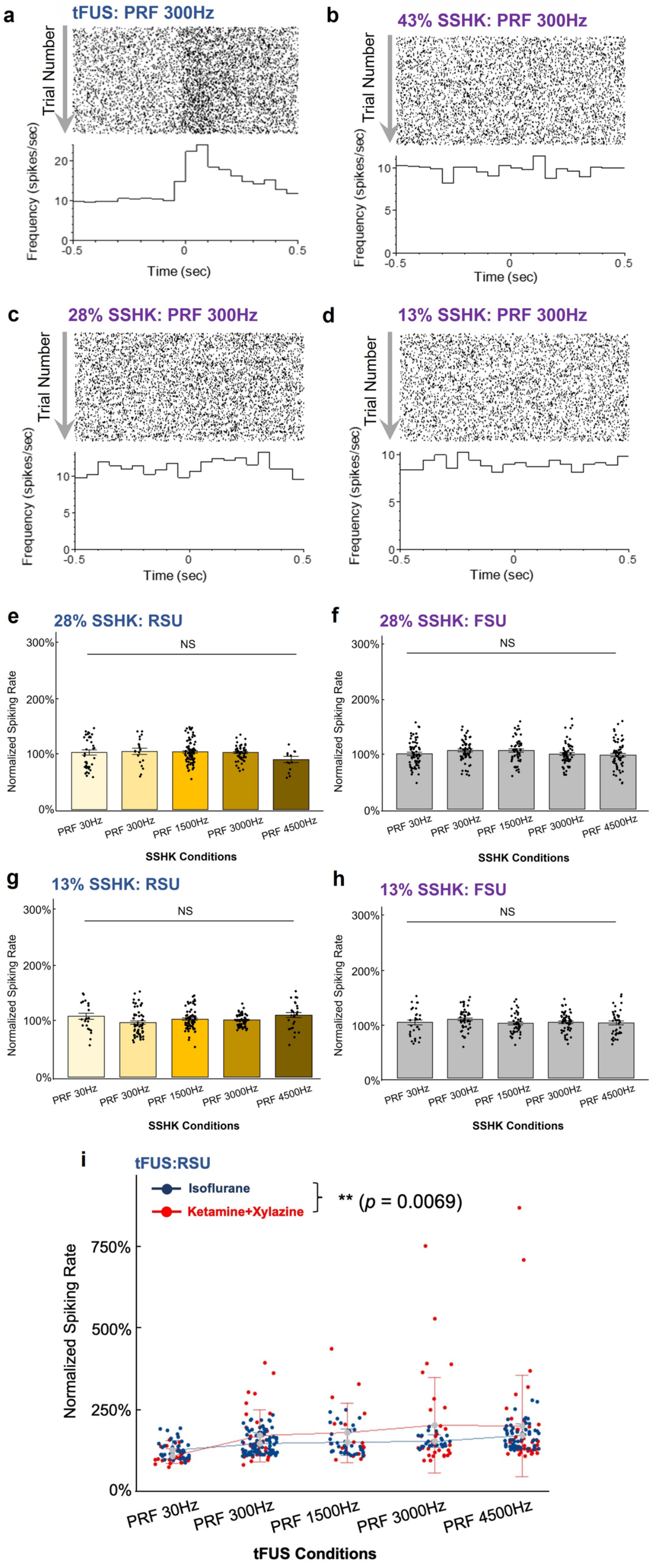

Supplementary Figure 6. Control for Potential Effects of Recording Electrode Vibration on Neuronal Activation, Related to Fig. 5.

(**a**-**d**) In order to control for mechanical vibration of the recording electrode leading to neural activation, control experiments were performed with tFUS delivered directly onto the electrode shank without transcranial delivery to the recording location shown in Fig. 1g and Fig. 5a. In the tFUS group, the experimental setup is shown in Fig. 1a. As an example, comparisons of spiking raster plots and PSTHs at PRF 300Hz show significantly different responses of the same neuron to tFUS stimulation (a) vs. to other three different levels of shank control experiments (b-d) (see more details about these levels in Methods).
(**e**-**h**) Statistical results of RSUs and FSUs responding to the PRF change in another two levels, i.e. 28% SSHK (209 RSUs, 232 FSUs) (e-f) and 13% (g-h) SSHK conditions (237 RSUs, 227 FSUs). No significant effect was observed through Kruskal-Wallis two-sided one-way ANOVA test.
(**i**) Statistical results of a 2-way ANOVA modeling the RSUs' (n = 144 for the ketamine+xylazine group, n = 245 for the isoflurane group) normalized responses to both the PRF change and the different anesthesia methods. The anesthesia method is observed to play a significant role (**p < 0.01) in changing the RSUs' spiking activities during the sonication period. Data are shown as the mean±s.e.m.. s.e.m., standard error of mean. Source data are provided in the Source Data file.

Supplementary Figure 7

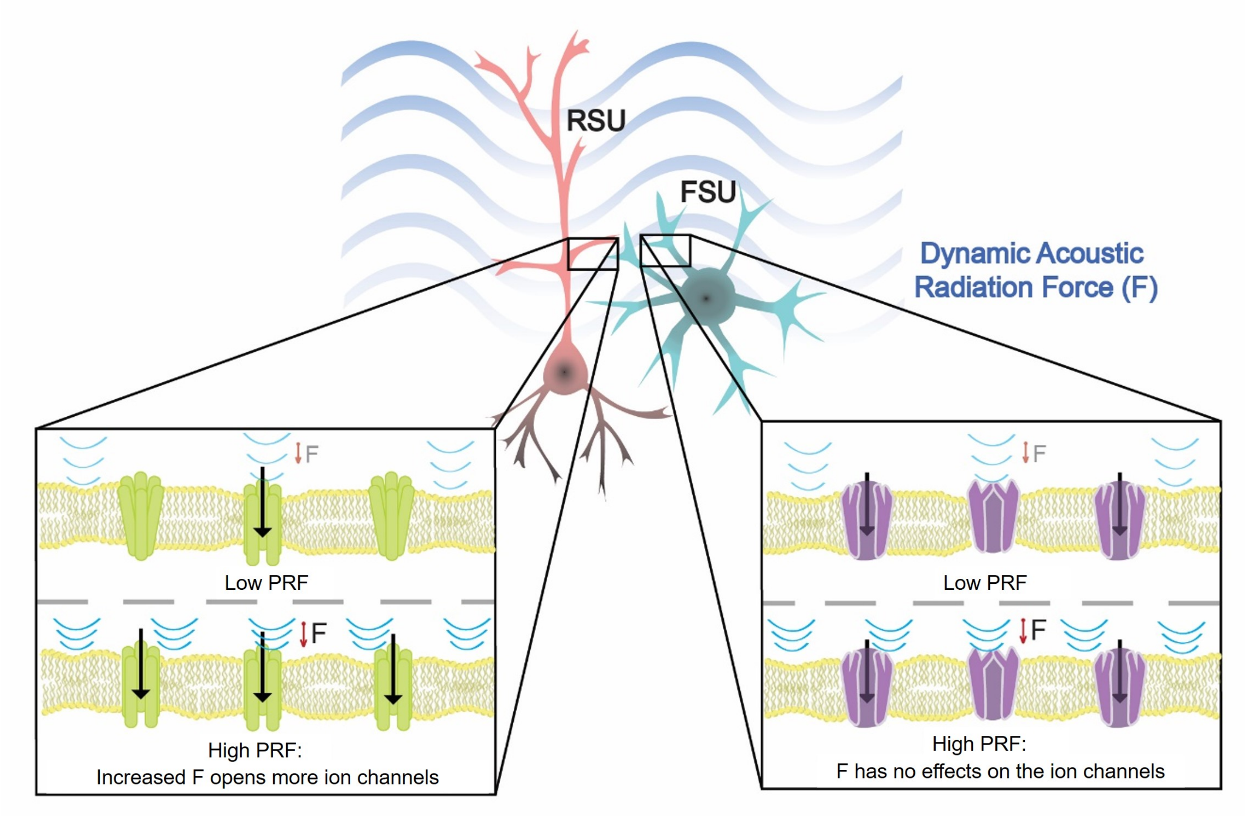

Supplementary Figure 7. Schematic Diagram Illustrates Hypothetical Differences of Types of Neurons in Response to the Dynamic Acoustic Radiation Force (ARF), Related to Discussion.

Note:

A hypothesized mechanism for observed difference in response to tFUS in distinct cell types. The dynamic acoustic radiation force is induced by the PRF. The RSUs exhibit more sensitivity to the ARF change than the FSUs do.

Supplementary Figure 8

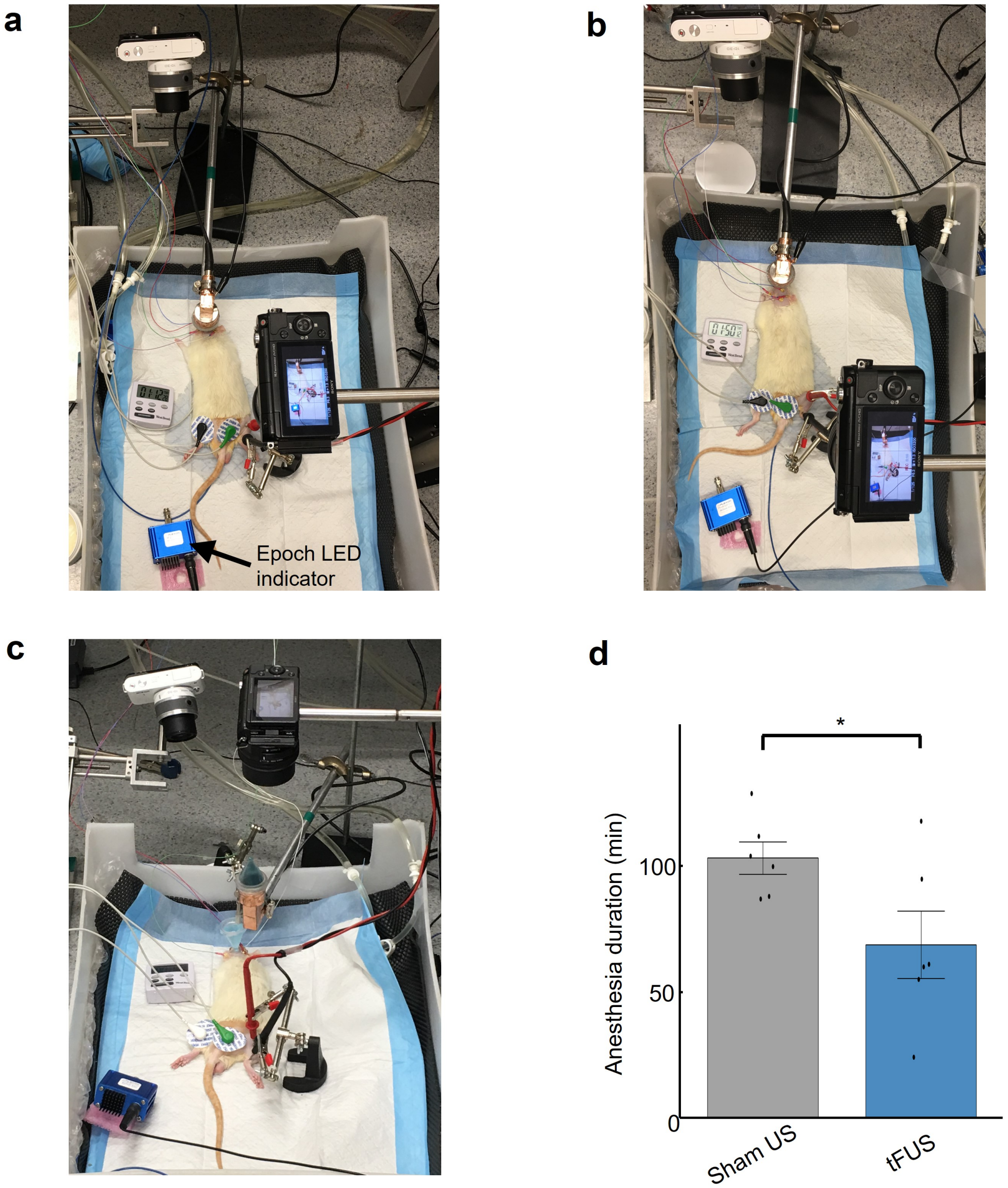

Supplementary Figure 8. tFUS Treatment Leads to Reduced Anesthesia Duration, Related to Discussion.

(**a**-**c**) Experimental setups for administering tFUS (a), SSKF (b), and electrical stimulation (c). Two cameras videotape after the anesthesia injection until an animal subject restores its moving capability.
(**d**) One-tail Wilcoxon test for a comparison between Sham US (n = 6 rats) (i.e. SSKF and electrical stimulation at contralateral hindlimb only) and tFUS (n = 6 rats) (Sham US vs. tFUS: $p = 0.046$). Data are shown as mean±s.e.m. *$p < 0.05$. Source data are provided in the Source Data file.

Supplementary Figure 9

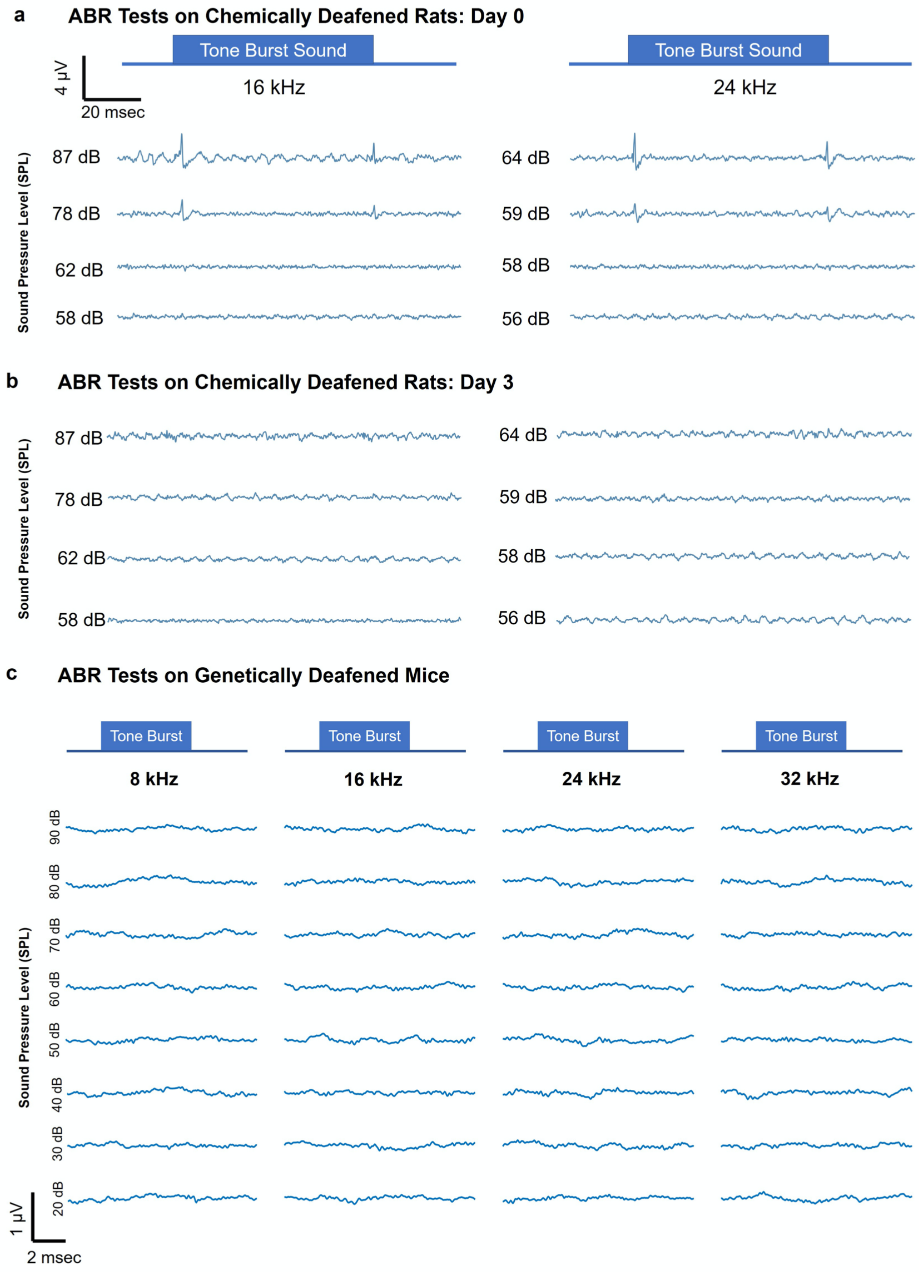

Supplementary Figure 9. Auditory Brainstem Response (ABR) Evidence from Deafened Rodents, Related to Discussion.
(**a-b**) ABR test results of rats before and after chemical injection. Four levels of sound intensity with 16 and 24 kHz center frequencies were used. On Day 3, post-injection ABR test results show significantly reduced auditory brainstem responses to the levels of sound intensities.
(**c**) ABR test results of genetically deafened mice. Genetically deaf naïve mice underwent tone burst stimulation. Eight levels of sound intensity with 8 to and 32 kHz center frequencies were used. Results show no significant auditory brainstem response to sound stimulus. Source data are provided in the Source Data file.

Supplementary Figure 10

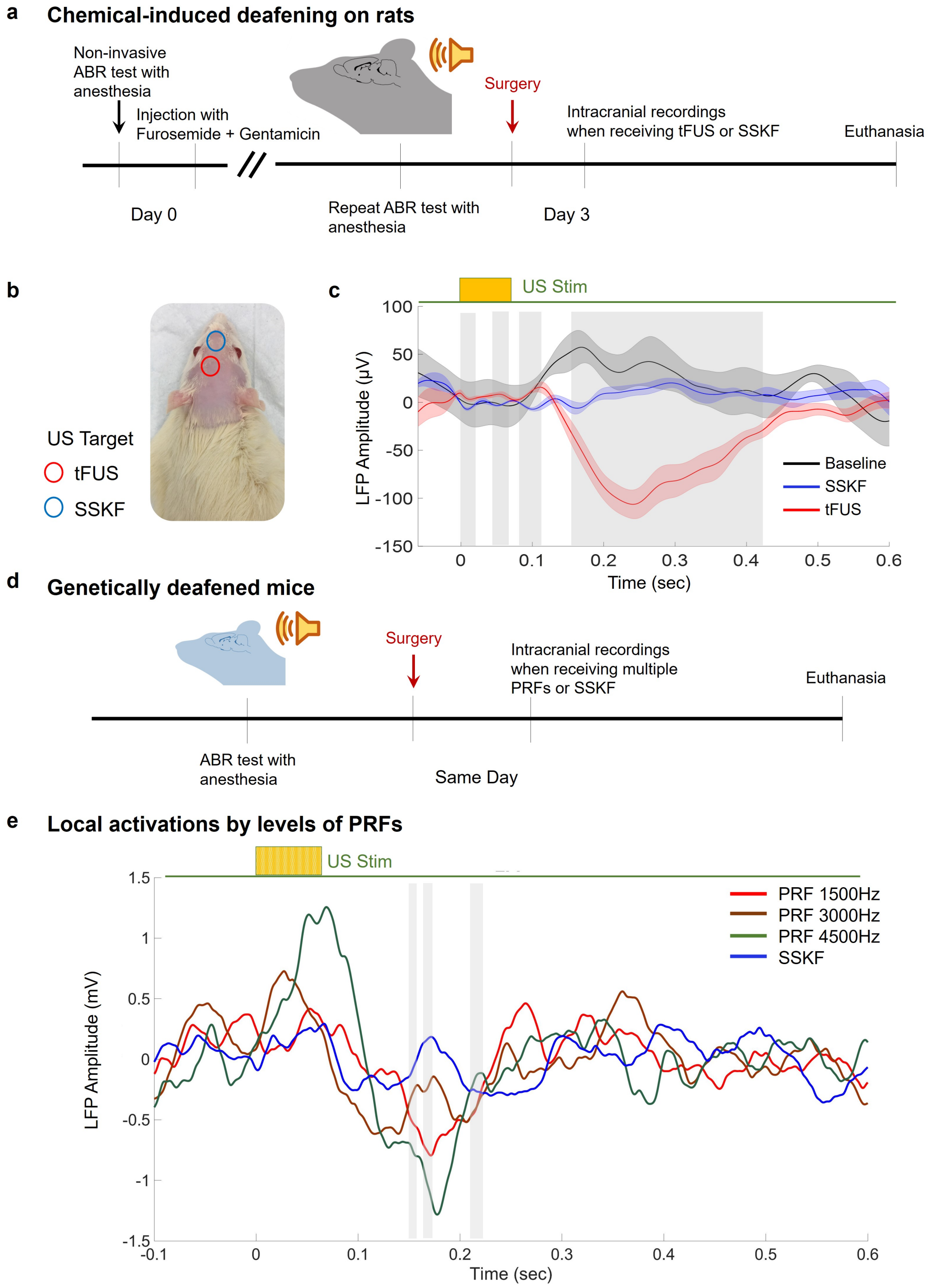

Supplementary Figure 10. tFUS Induced Local Brain Activity on Deafened Rodents (N = 5 in total), Related to Discussion.

(**a**) The experimental protocol for creating chemical-induced deafening rat model (N = 3) and neural recordings thereafter. The deafening will be tested by pre- and post-chemical injection auditory brainstem responses (ABR) tests on Day 0 and Day 3, respectively (Supplementary Fig. 9a-b). A combination of injected chemicals includes furosemide and gentamicin.

(**b**) On a photo representation of the rat in anesthesia after chemically deafened, ultrasound at the targeting location marked as red circle, or at the control location identified as blue.
(**c**) LFP recorded at the S1 area of a chemically deafened rat, averaged across 400 trials and presented with the mean value (solid line) and standard error of the mean (shaded areas). The employed ultrasound condition is PRF 1500Hz. Comparing with results from the sham condition, i.e. SSKF, significant differences with multiple comparison correction were observed and marked with vertical gray bars ($p < 0.05$). Two-sided parametric permutation test and false discovery rate (FDR) correction were made for the multiple comparisons.
(**d**) The experimental protocol for genetically deafened mice model (N = 2). The ABR tests will take place on the same day of electrophysiological recordings. The ARB results are presented in Supplementary Fig. 9c.
(**e**) LFP recorded at the S1 area of a genetically deafened mouse, averaged across 480 trials and presented with mean value. Three PRFs were introduced in order to compare the induced local brain activations with that from SSKF. The time-locked LFPs are evoked by the tFUS, but not by the SSKF. Comparing with results from the sham condition, significant differences with multiple comparison correction were observed and marked with vertical gray bars ($p < 0.05$). Two-sided parametric permutation test and FDR correction were made for the multiple comparisons. Source data are provided in the Source Data file.

Supplementary Figure 11

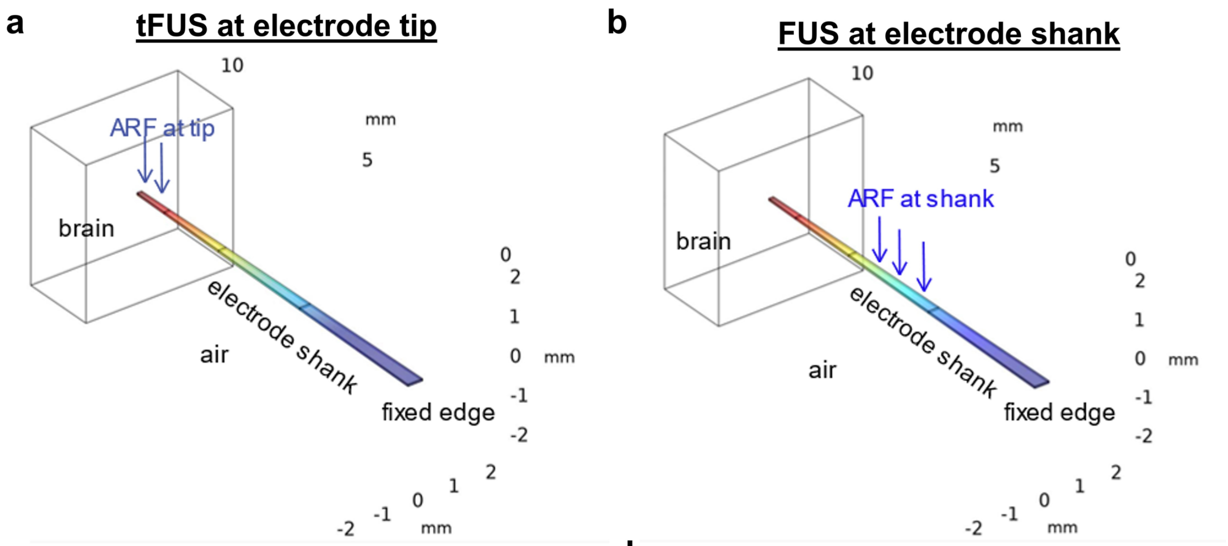

c

Ultrasound Conditions	ARF Magnitude (nN)	Max Displacement (nm)
**PRF 30Hz**	0.0081	0.0044
**PRF 300Hz**	0.081	0.045
**PRF 1500Hz**	0.41	0.22
**PRF 3000Hz**	0.81	0.45
**PRF 4500Hz**	1.22	0.67

d

Ultrasound Conditions	ARF Magnitude (nN)	Max Displacement (nm)
**PRF 30Hz**	0.49	0.02
**PRF 300Hz**	0.98	0.20
**PRF 1500Hz**	4.89	0.98
**PRF 3000Hz**	9.77	1.97
**PRF 4500Hz**	14.67	2.95

Supplementary Figure 11. Numerical Simulations of Microelectrode Array (MEA) As a Single Fixed-edge Cantilever Beam Inserted in Brain, Related to Discussion and Methods.

(**a-b**) Illustration of a single shank MEA electrode modeled as a single fixed-edge cantilever beam. The electrode was modeled as a silicon construct (density = 2330 kg/m^3, Young's modulus = 179 GPa) embedded in two fluids—brain tissue and air. The electrode width tapers from 0.4 mm at the fixed end to 0.125 mm at the tip. The electrode thickness is 0.05 mm. The electrode length is 10 mm of which 1 mm is in the brain and 9 mm is in air. Heat map illustrates calculated total displacement along the electrode shank. Simulated ARF is applied at locations shown by blue arrows as a uniform distributed force over the selected regions. (a) ARF uniformly applied at 1-mm segment at electrode tip. (b) ARF uniformly applied at 3-mm segment at midpoint of electrode shank.

(**c-d**) Calculated total ARF magnitude experienced by the electrode segment, used during simulation. ARF values are adjusted from Supplementary Table 1 based on the volume of the electrode segment experiencing ARF. Maximum displacement along the entire electrode is shown in (c-d). As observed in (a-b), maximum displacement is observed at the electrode tip in both simulated scenarios.

Supplementary Figure 12

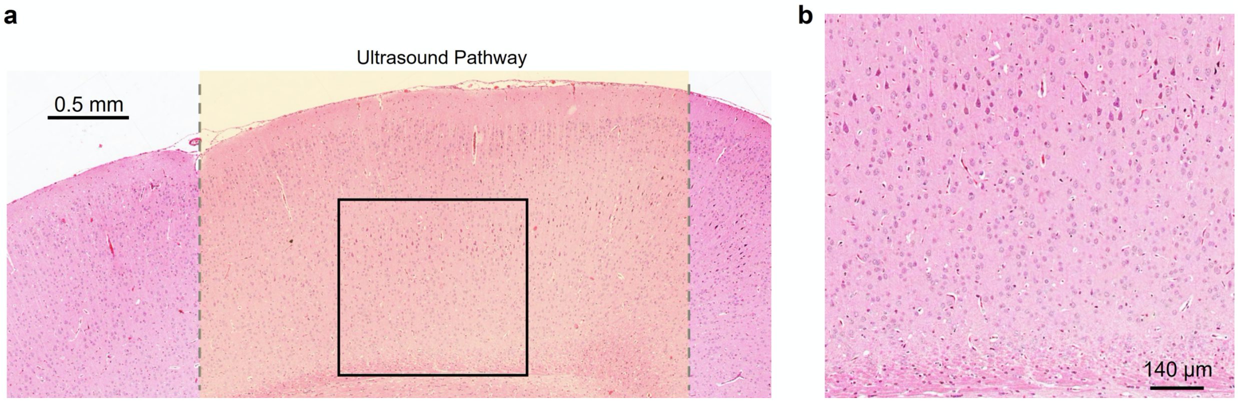

Supplementary Figure 12. tFUS Does Not Induce Neuron Damage, Local Hemorrhage or Inflammation, Related to Discussion and Methods.

H&E staining show no observable local lesion at somatosensory cortical region induced by tFUS. Physical size scales in each subplot are 500 and 140 µm, respectively. The ultrasound pathway is indicated with a yellow overlay. (**b**) is an enlarged version of the boxed region marked in (**a**). The histological images were repeated for 5 times independently with similar results.

Note:

In addition, the safety of the administered tFUS was also suggested in *in vitro* cell viability evaluation, where no significant difference was observed in the percentage of live/dead cells between the control and sonicated groups under 15 minutes stimulation at 4.5 kHz PRF[12]**.**

Supplementary Table 1. Estimations of Spatial-peak Acoustic Radiation Forces, Related to Discussion and Methods.

Ultrasound Conditions	Spatial Peak ARF magnitude (nN)
PRF 30Hz	10.9
PRF 300Hz	109.9
PRF 1500Hz	549.6
PRF 3000Hz	1099.2
PRF 4500Hz	1648.8

Supplementary Note 1: tFUS Shortens Anesthesia Recovery Time

In order to investigate the effect of tFUS to selectively target the somatosensory cortex, we perform control studies to test whether the perception of tFUS stimulation at the scalp can lead to cortical activations. Electrical stimulation at the hind limb is used as control for sensation.

Rat subjects

Naïve male rats around 12 weeks (body weight: median 386 g, range 370 – 400 g) were injected only with an initial dose of ketamine and xylazine cocktail (75, 10 mg/kg respectively) adjusted according to the animal weight. After sedation, rats are skin prepared and kept on heat pads to allow for recovery from anesthesia. Temperature, heart rate and video recording of movements are monitored.

Results

Recovery time is defined based on injection time and time of first perceived movement. Rats are divided in 3 subgroups. Group 1 (N = 6, Supplementary Fig. 8a) received tFUS stimulation (PRF 1500Hz) at S1HL, peripheral stimulation electrodes are set at the contralateral hindlimb with no current injection. Group 2 (N = 3, Supplementary Fig. 8c) received sham ultrasound directed 180 degrees away from the skull, only a collimator filled with ultrasound gel above S1HL, and peripheral stimulation electrodes are set at the contralateral hindlimb and monopolar pulse of peripheral stimulation at the contralateral hindlimb, current thresholds set at minimum current (1-3 mA dependent on subject, 0.08 – 0.1 msec set by PowerLab 26T) to induce a visible muscle contraction. Group 3 (N = 3, Supplementary Fig. 8b) received tFUS directed at the anterior part of the skull away from cortical regions of the brain (shown as in Fig. 1f and Supplementary Fig. 10b), and peripheral stimulation electrodes were set at the contralateral hindlimb with no current injection. We combined the Groups 2 and 3 as a Sham US group and then compared this new group to the Group 1. We found a significantly reduced anesthesia duration in the tFUS group, comparing to the Sham US group in which peripheral somatosensory inputs were applied by SSKF ultrasound or electrical stimulation.

Supplementary Note 2: Local Activations are Preserved in tFUS When Auditory Pathway is Blocked

In this study, in order to capture fast neuronal dynamics with high temporal resolution, we adopted intracranial electrophysiological recordings to directly study the neuronal responses at targeted brain locations. Recently, two published companion studies brought to attention the possibility of auditory confounding effects of ultrasound neuromodulation[1,4]. The findings of the companion studies call into attention confounding cortical activations that may arrive due to ultrasound mechanical coupling with the rodent skull. To control for these effects in our experimental setup, we have conducted control studies in chemically/genetically deafened rodents to ascertain that local activations are present in our setup without auditory percepts[5].

Auditory Brainstem Response (ABR) Test

ABR tests were performed on rats to determine the hearing threshold of rats before and after chemical deafening. Rodents were sedated under isoflurane (2%), 2 pairs of gold cup electrodes (Model 019-772500, Natus Medical Inc., Pleasanton, CA, USA) filled with EEG conductive paste (Ten20, Weaver and Company, Aurora, CO, USA) were applied to the rodent skull to record bilateral ABR[6]. Each pair of electrodes consists of one reference electrode at the forehead immediately below the left or right eye, and on the ipsilateral side, a recording electrode at the base of the ear. A common ground electrode was placed at the base of the skull between both ears. Each electrode pairs were placed in differential mode between the recording electrode and the reference electrode to determine the brainstem response to a particular sound stimulus. Rodents were placed inside insulated sound chambers, auditory stimuli were aligned with the ear, tone bursts lasting 67 msec (same duration as our ultrasound stimulus) were pulsed at 5 Hz for 750 trial at each frequency and amplitude combination. Stimulus were delivered with a 5-cm round frame cone speaker (16 Ohm, frequency response: 0.5-10 kHz) (Model 289-131, Parts Express, USA), and its output frequency controlled by a waveform generator (33612A, Keysight Technologies, Inc., USA) spanning between 8 to 24 kHz, amplitude ranging between 56 to 92 dB. For the ABR tests on genetically deafened mice, we conducted the ABR tests using a TDT system (128 Channel RZ2 Bioamp Processor with PZ5 Neurodigitizer Amplifier, Tucker Davis Technologies, USA).

Stimulation at Primary Somatosensory Cortex in Chemically Deafened Rats

To characterize whether neural responses observed are due to confounding effects of auditory stimulation, naïve animals received baseline ABR test prior to chemical deafening. Then, under

isoflurane anesthesia, rats received tail vein injection of furosemide at 175 mg/kg (purchased from Boynton Pharmacy, University of Minnesota) followed by subcutaneous injection of gentamicin at 350 mg/kg (purchased from VWR International)[7]. Post-injection ABR tests were performed 72 hours after initial injection. Once animals have been verified to have significant decrease in cochlear response, tFUS stimulation was applied at the somatosensory cortex, with concurrent intracranial recordings.

Stimulation at Primary Somatosensory Cortex in Genetically Deafened Mice

Adult mice subjects (*Atp2b2 or Pmca2*) older than P30 are used as subjects. Subjects are sedated initially under isoflurane (4% mixture with 1L/min O_2) in an induction chamber and extended with isoflurane (1.5% mixture with 0.2 L/min O_2) under a nose cone. Subject body temperature is stabilized with a heated water-bath, and vitals are monitored through ECG and respiration. tFUS collimator is coupled with the mice skull using ultrasound gel. Another ultrasound collimator with outlet aperture size of 2.3 mm was used to guide and transmit focused ultrasound onto mice scalp and through intact skull. The ultrasound was directed at a 30-degree angle towards the left S1 region of the mice brain.

Results

To test whether the induced neural activations is locally induced by tFUS, we performed control studies in chemically deafened rats under tFUS stimulation. The study methods are depicted as in Supplementary Fig. 10a. Auditory brainstem response (ABR) tests were conducted through subcutaneous needle EEG recordings to measure the rats' response to auditory stimuli, measured immediately before and 72-hour after chemical injections of a deafening cocktail of furosemide and gentamicin. Supplementary Fig. 9a shows the results from the ABR test of a naïve rat and Supplementary Fig. 9b shows a complete lack of response to auditory stimuli 3 days after the chemical injections. The deafening is conserved across different frequency ranges of auditory stimuli, an indication of widespread hearing loss.

After deafening is ascertained, tFUS was applied at S1, similar to experiments on healthy rats. Supplementary Fig. 10c illustrates the LFPs measured at S1 during baseline, tFUS stimulation and SSKF sham condition (PRF = 1500 Hz, I_{SPTA} = 52.74 mW/cm^2). Across 400 trials, time aligned activation is observed in the LFP during tFUS, originating at approximately 100 msec after tFUS onset (in contrast to the minimal delayed LFPs as in Supplementary Fig. 3 from wild-type rats without deafening drug administration). The standard error of mean potentials is illustrated; gray shaded regions show statistically significant differences amongst the average LFP traces. The

significantly different temporal regions are largely in the time segment of 155-417 msec after sonication, which is outside the timescale of auditory induced propagations observed in LFP data by one of the companion works[1]. This activation is significantly different compared to the sham condition (Supplementary Fig. 10b) at an anterior skull location tested through permutation-based non-parametric statistics with false discovery rate (FDR) multiple-comparison correction ($p < 0.05$).

To avoid potentially confounding damage to the cortical neural circuit due to the chemicals administered for deafening purpose, we applied the tFUS to the genetically deafened mice (see Supplementary Fig. 9c for ABR test result) following the experimental protocol illustrated in Supplementary Fig. 10d and changing the PRF levels listed in Table 1. As seen in Supplementary Fig. 10e for PRFs ranging from 1500 to 4500 Hz, the tFUS-induced LFPs exhibit an increasing trend in terms of peak-to-peak voltages. Without a functional auditory system, the *in vivo* brain still responds to the focused ultrasound energy at its targeted region, i.e. S1. However, no significant brain activations can be seen in the sham condition of SSKF when the ultrasound targeting at a similar anterior skull location of the mice subjects shown in Supplementary Fig. 10e.

Discussion

Based on a simultaneous EEG source imaging technique applied on rats reported previously[8], observations at the auditory cortex can be induced as a secondary activation when tFUS is not directed at the auditory cortex[5]. Therefore, in order to examine the auditory side effects[1,4] of the tFUS setup, presumably due to tFUS induced mechanical vibrations transmitted through the skull, a recent work on wild-type and deafened mice already showed strong evidence with motor and ABR measurements that the targeted brain activation is independent of peripheral auditory system[9]. Moreover, Yuan et al. applied low-intensity transcranial ultrasound to BALB/c mice explicitly reported new evidence for auditory-independent brain activations through multimodal readouts using optical imaging for cortical hemodynamic responses, electrophysiological recordings for LFPs and simultaneous motor activation[10]. To further understand the brain responses, we conducted tFUS stimulation after inducing permanently chemical deafening on rats and genetically deafening on mice (Supplementary Fig. 10a and d, respectively). Our observations of a significant rise (Supplementary Fig. 10c and e) in LFP at the S1 cortical region in the subject with significant reduction in hearing threshold level (Supplementary Fig. 9), suggest that side-effect activations in the auditory cortex from tFUS induced hearing percepts do not dictate activation of S1 cortex in our tFUS stimulation setup. Unsurprisingly, in these deafened models, the tFUS-induced LFP resembles the reported LFP waveform by Tufail et al.[11] on a mice model. Similar ultrasound parameters (e.g. f_0 and PRF) were employed in both studies (see

Supplementary Fig. 10c and the PRF 1500Hz in Supplementary Fig. 10e). In spite that the presence of the auditory pathways is disabled in the neural networks, significant tFUS activations can still be observed, providing direct evidence that our tFUS setup is able to induce local activations which are independent to the auditory perceptions.

Although the chemicals used in this group of experiments have been shown to damage auditory pathways, we do not have confirmation of whether these drugs present toxicity to other parts of the neural network. Therefore, one should be cautious in critically evaluating recordings gathered from these deafened rats. As presented in Supplementary Fig. 3, minimal delay in LFP activation (i.e. almost immediate response which is also seen in the PSTH presented in Fig. 3, Supplementary Fig. 5a and 6a) can be observed from the LFP profiles of naïve wild-type rats without undergoing chemical deafening procedures. Therefore, the time delay of the LFP data from chemical deafened rats (shown in Supplementary Fig. 10c) could be due to the administered chemical deafening agents. In order to control for auditory confounding effects leading to false positives in our tFUS stimulation in naïve rats, we performed two different sham conditions along with tFUS stimulation. To avoid potential side effects by introducing the deafening drugs, we also collected pilot data from genetically deafened mice to demonstrate the targeted brain activations (PRF = 1500, 3000 and 4500 Hz) when the auditory system is natively blocked (Supplementary Fig. 9c and 10e).

Supplementary Note 3: A Simulation of Electrode Vibrations During tFUS

We modeled the mechanical vibrations by the ultrasound and computed the potential displacement of electrode tip as shown in Supplementary Fig. 11. A COMSOL model was created to simulate electrode deflection properties while implanted in the brain and an ultrasound stimulation force is being applied. To model this scenario, we used the solid mechanics and fluid-structure interaction modules in COMSOL 5.5 (COMSOL, Inc., Burlington, MA). The electrode was modeled as a isotropic silicon construct (density = 2330 kg/m^3, Young's modulus = 179 GPa) embedded in two media—brain tissue and air. The electrode width tapers from 0.4mm at the fixed end to 0.125 mm at the tip. The electrode thickness is 0.05 mm. The electrode length is 10 mm of which 9 mm are in air and 1 mm is in the brain. The electrode fixed point or bending point is in the air fluid section. Dimensions of electrode are modeled after electrodes used during rat experiments (A1x32-Poly3-10mm-50-177, NeuroNexus, Ann Arbor, MI). To model the fluids, we encased the electrode beam in fluid areas extending 1 mm radially from beam center point in width and height. Due to electrode small dimensions, this volume is enough for the beam to be considered fully enclosed and we ignored any edge effects in the model. The brain tissue and air density properties are 1046 kg/m^3 and 1 kg/m^3 respectively. With the geometric conditions held constant, two scenarios were studied. In the first scenario, when tFUS is directed at the tip of the electrode, ARF is applied uniformly over the surface of 1 mm segment of electrode inserted into the brain. Total ARF magnitude used applied and calculated displacement are shown in Supplementary Fig. 11c. The electrode showed a maximum deflection at the tip of 0.67 nm or a 0.0013% deflection compared to the electrode thickness. In the second scenario, when tFUS is directed at the shank of the electrode, ARF is applied uniformly over a 3-mm segment of electrode in air centered at the electrode midpoint, area approximated by the -3 dB tFUS pressure profile. Total ARF magnitude used applied and calculated displacement are shown in Supplementary Fig. 11d. The electrode showed a maximum deflection at the tip of 2.95 nm or a 0.006% deflection compared to electrode thickness. The presented simulation ARF magnitude in Supplementary Fig. 11d accommodates adjusts for pressure difference across the skull, thus closely models that of SSHK 43% condition. Our results indicate the maximum electrode displacement occurs at the tip of the electrode. Moreover, when tFUS is applied at the shank of the electrode in SSHK 43%, the resulting displacement is greater than typical experimental conditions, when tFUS is applied at the electrode tip. Therefore, our simulations suggest that SSHK 43% is an adequate sham condition to examine the effect of electrode vibration on neural activation.

Supplementary References:

1 Guo, H. *et al.* Ultrasound Produces Extensive Brain Activation via a Cochlear Pathway. *Neuron* **99**, 866, doi:10.1016/j.neuron.2018.07.049 (2018).

2 Kim, H., Chiu, A., Lee, S. D., Fischer, K. & Yoo, S. S. Focused ultrasound-mediated non-invasive brain stimulation: examination of sonication parameters. *Brain Stimul* **7**, 748-756, doi:10.1016/j.brs.2014.06.011 (2014).

3 Treeby, B. E. & Cox, B. T. k-Wave: MATLAB toolbox for the simulation and reconstruction of photoacoustic wave fields. *Journal of biomedical optics* **15**, 021314, doi:10.1117/1.3360308 (2010).

4 Sato, T., Shapiro, M. G. & Tsao, D. Y. Ultrasonic Neuromodulation Causes Widespread Cortical Activation via an Indirect Auditory Mechanism. *Neuron* **98**, 1031-1041 e1035, doi:10.1016/j.neuron.2018.05.009 (2018).

5 Niu, X., Yu, K. & He, B. On the neuromodulatory pathways of the in vivo brain by means of transcranial focused ultrasound. *Current Opinion in Biomedical Engineering* **8**, 61-69, doi:https://doi.org/10.1016/j.cobme.2018.10.004 (2018).

6 Akil, O., Oursler, A. E., Fan, K. & Lustig, L. R. Mouse Auditory Brainstem Response Testing. *Bio Protoc* **6**, doi:10.21769/BioProtoc.1768 (2016).

7 McGuinness, S. L. & Shepherd, R. K. Exogenous BDNF rescues rat spiral ganglion neurons in vivo. *Otol Neurotol* **26**, 1064-1072 (2005).

8 Yu, K., Sohrabpour, A. & He, B. Electrophysiological Source Imaging of Brain Networks Perturbed by Low-Intensity Transcranial Focused Ultrasound. *IEEE Trans Biomed Eng* **63**, 1787-1794, doi:10.1109/TBME.2016.2591924 (2016).

9 Mohammadjavadi, M. *et al.* Elimination of peripheral auditory pathway activation does not affect motor responses from ultrasound neuromodulation. *Brain Stimulation*, doi:https://doi.org/10.1016/j.brs.2019.03.005 (2019).

10 Yuan, Y., Wang, Z., Liu, M. & Shoham, S. Cortical hemodynamic responses induced by low-intensity transcranial ultrasound stimulation of mouse cortex. *NeuroImage* **211**, 116597, doi:10.1016/j.neuroimage.2020.116597 (2020).

11 Tufail, Y. *et al.* Transcranial Pulsed Ultrasound Stimulates Intact Brain Circuits. *Neuron* **66**, 681-694, doi:DOI 10.1016/j.neuron.2010.05.008 (2010).

12 DeBari, M., Niu, X., Pereia, S., Griffin, M., He, B., and Abbott, R. Therapeutic Ultrasound Triggered Silk Scaffold Degradation for Tissue Regenerative Applications, in *2019 Biomedical Engineering Society Annual Meeting*, Philadelphia, October 16-19, 2019.
