# Supplementary material for: Intrinsic functional neuron-type selectivity of transcranial focused ultrasound neuromodulation
Source: Nat Commun. 2021 May 4;12:2519. doi: 10.1038/s41467-021-22743-7 (PMC8097024; doi:10.1038/s41467-021-22743-7)
Supplement: Supplementary file 3 — Reporting Summary [file 41467_2021_22743_MOESM3_ESM.pdf]

nature research

Corresponding author(s): Bin He

Last updated by author(s): 03/03/2021

Reporting Summary

Nature Research wishes to improve the reproducibility of the work that we publish. This form provides structure for consistency and transparency in reporting. For further information on Nature Research policies, see our Editorial Policies and the Editorial Policy Checklist.

Statistics

For all statistical analyses, confirm that the following items are present in the figure legend, table legend, main text, or Methods section.

n/a	Confirmed	
☐	☒	The exact sample size (*n*) for each experimental group/condition, given as a discrete number and unit of measurement
☐	☒	A statement on whether measurements were taken from distinct samples or whether the same sample was measured repeatedly
☐	☒	The statistical test(s) used AND whether they are one- or two-sided<br>*Only common tests should be described solely by name; describe more complex techniques in the Methods section.*
☐	☒	A description of all covariates tested
☐	☒	A description of any assumptions or corrections, such as tests of normality and adjustment for multiple comparisons
☐	☒	A full description of the statistical parameters including central tendency (e.g. means) or other basic estimates (e.g. regression coefficient) AND variation (e.g. standard deviation) or associated estimates of uncertainty (e.g. confidence intervals)
☐	☒	For null hypothesis testing, the test statistic (e.g. *F*, *t*, *r*) with confidence intervals, effect sizes, degrees of freedom and *P* value noted<br>*Give P values as exact values whenever suitable.*
☒	☐	For Bayesian analysis, information on the choice of priors and Markov chain Monte Carlo settings
☒	☐	For hierarchical and complex designs, identification of the appropriate level for tests and full reporting of outcomes
☒	☐	Estimates of effect sizes (e.g. Cohen's *d*, Pearson's *r*), indicating how they were calculated

Our web collection on statistics for biologists contains articles on many of the points above.

Software and code

Policy information about availability of computer code

Data collection	Intracranial data collection used NeuroNexus 32 channel poly-3 recording electrodes and opto-electrodes. ABR recordings are collected with needle EEG electrodes. Ex-vivo pressure recordings are collected with needle hydrophone HNR500 and National Instruments DAQ box. NeuroNexus smartbox and TDT RZ2 systems are used for acquiring neural data. The ADInstruments PowerLab 26T system is used to monitor the vital signals of rodents during the in vivo experiments. Details about data collection are included in the Methods.
Data analysis	Spike sorting is performed using Offline Sorter and further analyzed with open source package FieldTrip Toolbox (Version 20170508). All other data processing is performed in MATLAB (V9.0.0) with wavelet denoising performed in Wavelet Toolbox (V4.16). Statistical analyses are performed with both FieldTrip Toolbox in MATLAB (V9.0.0) and in R (V3.2.1 and V3.5.3). Simulations performed in COMSOL (V5.5) and K-wave (V1.1). The normalized neuronal spiking activities were analyzed with custom code, which has been deposited to FigShare (https://doi.org/10.6084/m9.figshare.14150336.v1).

For manuscripts utilizing custom algorithms or software that are central to the research but not yet described in published literature, software must be made available to editors and reviewers. We strongly encourage code deposition in a community repository (e.g. GitHub). See the Nature Research guidelines for submitting code & software for further information.

Data

Policy information about availability of data

All manuscripts must include a data availability statement. This statement should provide the following information, where applicable:

- Accession codes, unique identifiers, or web links for publicly available datasets
- A list of figures that have associated raw data
- A description of any restrictions on data availability

The data that support the findings of this study are presented in the paper and supplementary materials. Source data are provided with this paper. Data of sorted

neural spikes in 10 animals can be found from Figshare repository at: doi.org/10.6084/m9.figshare.14150336. Additional data are available from the corresponding author upon reasonable request.

Field-specific reporting

Please select the one below that is the best fit for your research. If you are not sure, read the appropriate sections before making your selection.

☒ Life sciences ☐ Behavioural & social sciences ☐ Ecological, evolutionary & environmental sciences

For a reference copy of the document with all sections, see nature.com/documents/nr-reporting-summary-flat.pdf

Life sciences study design

All studies must disclose on these points even when the disclosure is negative.

Sample size	Sample sizes were determined based on previous studies (Murray and Keller, 2011; Tufail, et al, 2010; Yoo, et al, 2011, etc.) to make sure that experiments can be replicated to ensure confidence in the results. Sample sizes are mentioned in the Statistical Methods subsection.
Data exclusions	Recordings with confirmed misplacement of stimulation/recording location or failed surgery were excluded from the dataset.
Replication	The experiments were performed independently to 3-10 replicates per experiment condition. Aside from data exclusions, all replications are successful.
Randomization	Subjects were randomly selected for experimental conditions and subject groups. If multiple conditions applied, those conditions are randomized in order as described in the Discussion section.
Blinding	To minimize bias, the intrinsic cell-type selectivity data from transgenic mice preprocessing and statistical analyses were conducted by separate designated authors. The authors for each step were blinded to the experimental condition.<br>Data from other in vivo experiments were acquired and analyzed without experimenter blinding, because given the all-day long experiment on rat, the experimenters need to alternatively operate and monitor the experiments, e.g., ensuring the anesthesia depth, adjusting the stimulation paradigms and monitoring the recording quality. Therefore, in these in vivo experiments, we were not able to perform total blinding.

Reporting for specific materials, systems and methods

We require information from authors about some types of materials, experimental systems and methods used in many studies. Here, indicate whether each material, system or method listed is relevant to your study. If you are not sure if a list item applies to your research, read the appropriate section before selecting a response.

Materials & experimental systems

n/a	Involved in the study
☒	☐ Antibodies
☒	☐ Eukaryotic cell lines
☒	☐ Palaeontology and archaeology
☐	☒ Animals and other organisms
☒	☐ Human research participants
☒	☐ Clinical data
☒	☐ Dual use research of concern

Methods

n/a	Involved in the study
☒	☐ ChIP-seq
☒	☐ Flow cytometry
☒	☐ MRI-based neuroimaging

Animals and other organisms

Policy information about studies involving animals; ARRIVE guidelines recommended for reporting animal research

Laboratory animals	Rat subjects used are adult Wistar outbred rats (Hsd:WI purchased from Envigo, age: 2-4 month). All rat subjects were male to control for effects of menstrual cycle hormones on neural activity.<br>Genetically deaf mice subjects C3H/HeJ-Atp2b2<dfw>/J were purchased from Jackson Labs (age: 1-4 month). Due to low frequencies of desired genotypes, both male and female mice are used.<br>Optogenetic mice subjects used are adult Black 6 based transgenic models (B6.Cg-Tg(Camk2a-cre)T29-1Stl/J, B6;129P2-Pvalbtm1(cre)Arbr/J, B6;129S-Gt(ROSA)26Sortm32.1(CAG-COP4*H134R/EYFP)Hze/J purchased from Jackson Labs, age: 1-4 month). Due to low frequencies of desired genotypes, both male and female mice are used.
Wild animals	Study did not involve wild animals.
Field-collected samples	Study did not involve field-collected samples.
Ethics oversight	The Institutional Animal Care and Use Committees (IACUC) at Carnegie Mellon University and the University of Minnesota oversee

the ethical considerations of this project. All protocols and procedures were approved by the IACUCs.

Note that full information on the approval of the study protocol must also be provided in the manuscript.
